# Uncovering distinct predictors of diabetes distress and depressive symptoms in a longitudinal survival analysis of incidence and remission: indication for diverging aetiological paths

**DOI:** 10.1007/s00125-026-06666-w

**Published:** 2026-01-22

**Authors:** Gina Lehmann, Dominic Ehrmann, Birgit Olesen, Lilli-Sophie Priesterroth, Bernhard Kulzer, Thomas Haak, Norbert Hermanns

**Affiliations:** 1 Research Institute Diabetes Academy Mergentheim (FIDAM), Bad Mergentheim, Germany; 2 Department of Clinical Psychology and Psychotherapy, University of Bamberg, Bamberg, Germany; 3 Health Psychology, Institute of Psychology, Johannes Gutenberg-University Mainz, Mainz, Germany; 4 Diabetes Center Mergentheim, Bad Mergentheim, Germany

**Keywords:** Anxiety disorder, Depression, Diabetes distress, Incidence, Longitudinal, Remission, Survival analysis

## Abstract

**Aims/hypothesis:**

The aim of the study was to examine the course of diabetes distress and depressive symptoms and their predictors of incidence and remission in individuals with type 1 and type 2 diabetes.

**Methods:**

Data were collected every 6 months over a 24 month period. Participants (*n*=654) completed measures of diabetes distress (Problem Areas in Diabetes Scale) and depressive symptoms (Patient Health Questionnaire 8). Cox proportional hazards models were applied to examine predictors of incidence and remission, considering demographic, clinical and psychosocial factors. All predictors were assessed at baseline, except for HbA_1c_, which was modelled as a time-varying covariate.

**Results:**

Diabetes distress showed cumulative incident cases in 21% of the sample and a remission rate of 70% across 24 months. For depressive symptoms, cumulative 24 month incidence was 33% and remission was 67%. The median onset time was 18 months for diabetes distress and 24 months for depressive symptoms; the median remission time for both was 6 months. Higher HbA_1c_ (HR=1.02, *p*=0.022), female gender (male gender HR=0.55, *p*=0.043), long-term complications (HR=2.11, *p*=0.009) and a history of anxiety disorders (HR=2.57, *p*=0.029) significantly predicted the incidence of diabetes distress, while no predictors were associated with remission. For depressive symptoms, higher HbA_1c_ (HR=1.03, *p*<0.001), prior depression (HR=2.63, *p*=0.001) and eating disorders (HR=2.20, *p*=0.044) predicted incidence. Remission was significantly associated only with older age (HR=1.02, *p*=0.045).

**Conclusions/interpretation:**

Suboptimal glycaemic outcomes predicted both diabetes distress and depression; however, diabetes distress was associated with anxiety disorders, whereas depressive symptoms were linked to prior depression and eating disorders, hinting at distinct aetiologies.

**Graphical Abstract:**

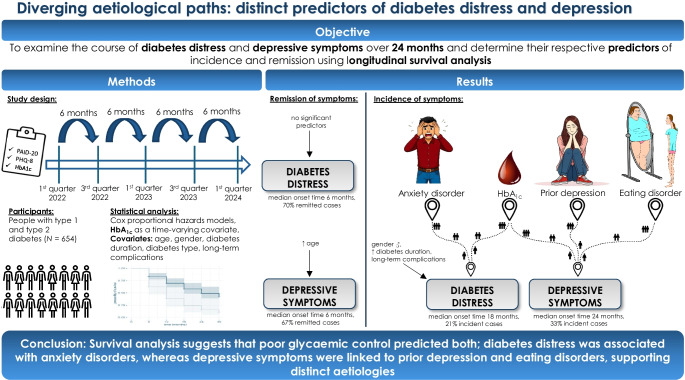

**Supplementary Information:**

The online version contains peer-reviewed but unedited supplementary material available at 10.1007/s00125-026-06666-w.


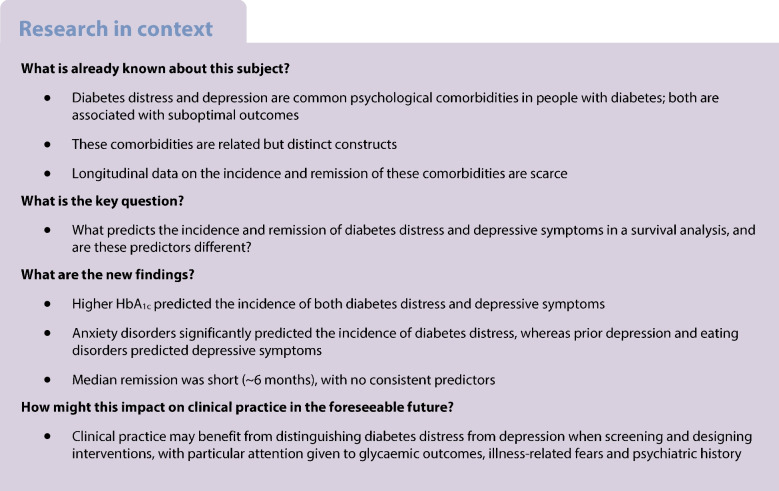


## Introduction

There is hardly any chronic disease that requires as much discipline in self-care as the chronic metabolic condition diabetes. It is therefore not surprising that many individuals report feeling mentally exhausted following the sudden change in lifestyle after diagnosis and the high demands of dealing with the condition [1]. As a consequence, diabetes-specific burdens, commonly referred to as diabetes distress, are rather frequent [2, 3]. A meta-analysis found a prevalence of 36% in people with type 2 diabetes [4]. In type 1 diabetes, diabetes distress prevalence varies between studies, but according to Skinner et al [2] it ranges from 20% to 40%.

Research has consistently shown that diabetes distress is linked to impaired physical and mental health. It is associated with suboptimal glycaemic management [3, 5–9], a greater prevalence of long-term complications [7] and lower quality of life [10]. Higher levels of distress have been reported among younger individuals, women and those with complications [11, 12]. Distress levels are often highest shortly after diagnosis [2].

The prevalence of affective disorders like depression and anxiety is also elevated in people with diabetes [13]. Farooqi et al [13] reported an overall depression prevalence of 19% among people with diabetes, with rates of 22% in type 1 and 19% in type 2 diabetes (almost twice as high as in people without diabetes). Depressive symptoms are linked to reduced quality of life [14], suboptimal diabetes self-management [15] and a higher prevalence of long-term complications [16, 17]. Women with diabetes are at higher risk [18–20].

Diabetes distress and depressive symptoms share overlapping features, such as feelings of hopelessness, reduced motivation and emotional exhaustion. However, they represent related yet conceptually distinguishable constructs. Diabetes distress specifically reflects the emotional burden of managing diabetes and its treatment demands, whereas depression is a broader and more persistent mood disturbance that is not necessarily tied to diabetes itself [3, 21].

Given the strong associations of diabetes distress and depressive symptoms with clinical and psychosocial outcomes, addressing these burdens is essential in diabetes care [22]. Longitudinal studies are particularly important for identifying the risk and protective factors that influence mental health trajectories over time and for evolving targeted prevention and intervention strategies.

Although many cross-sectional studies have examined the prevalence and correlates of diabetes distress and depressive symptoms, few have investigated longitudinal relationships with psychosocial outcomes. Most studies have used regression and latent growth models [23–27]. For instance, Fisher et al [23] used logistic regression analyses to identify risk factors for elevated diabetes distress in people with type 2 diabetes and found that being female, having a prior history of major depressive disorder, experiencing more negative life events, having more diabetes complications and maintaining a poor diet and low physical activity increased the odds of becoming distressed over time. In a longitudinal analysis with people with type 1 diabetes, Fisher et al [25] reported that age, gender and the number of complications predicted changes in distress levels over time. Associations with greater risk of persistent depressive symptoms were younger age, female gender and a lifetime history of major depressive disorder [27]. While these studies provided valuable insights, survival analysis offers additional methodological advantages such as modelling the relationship between predictors and the timing of an event; however, it has rarely been applied in this context.

De Groot et al [28] applied survival analysis to estimate the duration, recurrence and remission of depressive episodes in individuals with type 2 diabetes. They reported a median duration of up to 14 months for initial episodes and showed that remission periods became progressively shorter with each subsequent episode. Similarly, Nefs et al [29] examined the longitudinal course of depressive symptoms in a large cohort of 2460 primary care patients with type 2 diabetes, showing that depressive symptoms persisted or recurred over time in 66% of the sample. However, survival analysis has rarely been applied to study such trajectories and, to the best of our knowledge, no previous study has used this method to examine predictors of diabetes distress onset or remission [30, 31].

The present study addresses this gap by applying Cox proportional hazards models to identify risk and protective factors related to the onset and remission of diabetes distress and depressive symptoms over time. Furthermore, we aim to model the temporal course of these conditions and provide clinically relevant insights into when these conditions are most likely to emerge or subside.

## Methods

### Study procedure

The data were collected via the online panel dia·link (https://www.dialink-diabetes.de). The panel is for people with diabetes, their relatives and practitioners from the field of diabetology, who can register for free to take part in regular surveys on diabetes-specific issues. We assume that the panel members are likely educated, primarily German-speaking adults with a high degree of engagement in their diabetes management, who are interested in research and motivated to contribute. All surveys are conducted online, and informed consent is obtained before each survey. For this study, eligible panellists (≥18 years of age with type 1 or type 2 diabetes) received a notification via email every 6 months and were asked to take part in a survey about their quality of life with diabetes. Participants filled in various questionnaires regarding their mental health, including diabetes distress and depressive symptoms. The first survey took place in the first quarter of 2022. So far, four more surveys have been collected, resulting in a total of five points of measurement over a time span of 24 months. As the survey was conducted on a voluntary basis, not everyone took part in all five surveys and some also joined in later, which resulted in a slight fluctuation in the number of participants at every point of measurement. For this analysis, we included those who had at least two points of measurement.

### Ethical standard

The study was approved by the Ethics Committee of the German Psychological Society (DGPs) (file HermannsNorbert2020-12-18 VA) and carried out in accordance with the Declaration of Helsinki. All participants provided written informed consent before inclusion.

### Variables and measures

At baseline, which is individually defined as the first time a person entered the survey, we obtained self-reported demographic variables, including age and gender (female, male or diverse), as well as medical data, such as diabetes duration, type of diabetes, long-term complications such as retinopathy, neuropathy, nephropathy and diabetic foot syndrome, and a history of diagnosed psychological comorbidities such as major depression, eating disorder and anxiety disorders. Ethnicity data were not collected, as we assume that the participants of the dia·link panel are primarily German-speaking adults without diverse ethnic backgrounds. Starting from a participant’s individual baseline, self-reported HbA_1c_ levels (which are typically measured every 3 months as part of standard diabetes care) and mental health aspects such as diabetes distress and depressive symptoms were measured every 6 months.

Diabetes distress was assessed using the German version of the Problem Areas in Diabetes Scale (PAID) [7]. The questionnaire requests ratings of 20 diabetes-specific emotional problems (e.g. fear of long-term complications, problems with hypoglycaemia and overburdening due to diabetes treatment) on a five-point Likert scale from 0 (‘not a problem’) to 4 (‘serious problem’). The item scores were multiplied by 1.25, resulting in a total score ranging from 0 to 100, with higher scores suggesting higher diabetes distress. Total scores of 40 or higher are considered as clinically elevated distress [32] and therefore were used as the cut-off.

Depressive symptoms were assessed using the Patient Health Questionnaire 8 (PHQ-8). The PHQ-8 is a reliable instrument for recording the frequency and severity of depressive moods [33] and has also been proven to be a suitable method for detecting depression in people with diabetes [34]. The questionnaire consists of eight items based on the diagnostic criteria of the fifth edition of the Diagnostic and Statistical Manual of Mental Disorders (DSM-V) for depression. On a four-point Likert scale from 0 (‘not at all’) to 3 (‘almost every day’), participants were asked to indicate how often they felt affected by the complaints (e.g. ‘little interest or pleasure in your activities’ or ‘tiredness or feeling of having no energy’). The scores of the eight items were summed to obtain a value range of 0–24. A cut-off value of 10 indicated the presence of a depressive mood [33].

### Statistical analyses

The statistical analyses were performed using IBM SPSS Statistics 29 (https://www.ibm.com/products/spss-statistics) and R Statistical Software (https://www.r-project.org/, v4.3.3) [35].

The prevalence of elevated diabetes distress and depressive symptoms was calculated at each of the five measurement points as the proportion of individuals with a PAID score ≥40 and a PHQ score ≥10, respectively. These cut-off values were also used for computing the cumulative incidence and remission rates.

Cox proportional hazards models were applied to identify factors influencing the onset and remission of diabetes distress and depressive symptoms. As with conventional linear regression models, survival regression models allow the effects of covariates and predictor variables to be investigated. However, Cox proportional hazards models characterise how covariates relate to the time until a specific event occurs, by modelling how hazard changes over time [36].

The key factor in this analysis is the time between a defined start and the point at which the event of interest occurs, commonly referred to as ‘time-to-event’ [37]. The ‘event’ variable indicates whether the event has occurred (e.g. if a value exceeds a cut-off) and was dichotomised as 0 (‘event has not [yet] occurred’) and 1 (‘event has occurred’). This allows for inclusion of both observed events and censored cases (i.e. when the event has not occurred by the end of the study). Events can be negative (incidence of symptoms: PAID ≥40 or PHQ ≥10) or positive (remission: PAID <40 or PHQ <10) at any of the points of measurement. For incidence analyses, we excluded individuals who already reported elevated symptoms at their individual first point of measurement; for remission analyses, we included only those with elevated symptoms at their individual baseline.

In the present dataset, demographic and clinical variables (age, diabetes duration, diabetes type and HbA_1c_), as well as the presence of pre-existing long-term complications and a history of psychological comorbidities (depression, eating disorder and anxiety), were included as predictors in the model. Gender was additionally entered as a covariate in all Cox proportional hazards models to examine potential gender-related differences. As no participant identified as ‘diverse’, analyses were limited to female and male participants. All predictors were assessed at baseline, except for HbA_1c_, which was modelled as a time-varying covariate corresponding to the same time point as each assessment to account for dynamic changes in glycaemic outcomes. Therefore, we generated a data frame in long format with each row corresponding to a follow-up time interval with the respective covariate value.

The proportional hazards assumption, a key requirement of the Cox regression model, states that the hazard ratios for the covariates remain constant over time. To test this assumption, we used the Schoenfeld residuals and visually inspected residual plots to identify any systematic time-dependent patterns. There was no significant deviation for all variables included. Multicollinearity was examined by inspecting variance inflation factors and tolerance values, which indicated no problematic correlations among predictors.

A* p* value <0.05 (two-tailed) was considered to indicate statistical significance in all analyses.

## Results

### Sample characteristics

The study sample consisted of 654 individuals, who participated in at least two points of measurement. This sample did not significantly differ from the full sample size (*n*=1209) in terms of gender, HbA_1c_, diabetes type and diabetes duration (all *p*>0.05). Participants who completed at least two assessments were on average about 5 years older than those who participated only once (*p*<0.001). For the incidence analyses, only individuals without elevated symptoms at their baseline were included (*n*=501 for diabetes distress, *n*=486 for depressive symptoms). Participants who reported elevated symptoms at their individual baseline (*n*=131 for diabetes distress, *n*=168 for depressive symptoms) were included in the remission analyses. The mean age of participants was 55.4 years (SD=13.9, range=17–85). The mean duration of diabetes was 25.8 years (SD=15.8, range=3–71). The mean HbA_1c_ was 55 mmol/mol (7.2±1.1%). In terms of gender distribution, 48.5% were female and 51.5% were male. The majority had type 1 diabetes (76.0%). Furthermore, 22.2% of participants had a history of diagnosed depression, 11.3% had a history of diagnosed anxiety disorders and 7.5% had a history of diagnosed eating disorders. For full sample characteristics, see Table 1.

**Table 1:** Sample characteristics

Variable	Mean ± SD (range) or *n* (%)
Age, years	55.4±13.9 (17–85)
Diabetes duration, years	25.8±15.8 (3–71)
HbA_1c_, mmol/mol, baseline	55
HbA_1c_, %, baseline	7.2±1.1
HbA_1c_, mmol/mol, 6 months	52
HbA_1c_, %, 6 months	6.9±0.9
HbA_1c_, mmol/mol, 12 months	52
HbA_1c_, %, 12 months	6.9±0.9
HbA_1c_, mmol/mol, 18 months	52
HbA_1c_, %, 18 months	6.9±0.9
HbA_1c_, mmol/mol, 24 months	51
HbA_1c_, %, 24 months	6.8±0.8
Male gender	337 (51.5)
Type 1 diabetes	497 (76.0)
Pre-existing long-term complications	251 (38.4)
History of diagnosed depression	145 (22.2)
History of diagnosed anxiety	74 (11.3)
History of diagnosed eating disorder	49 (7.5)

### Incidence and remission rates of diabetes distress and depressive symptoms

**Fig. 1:**
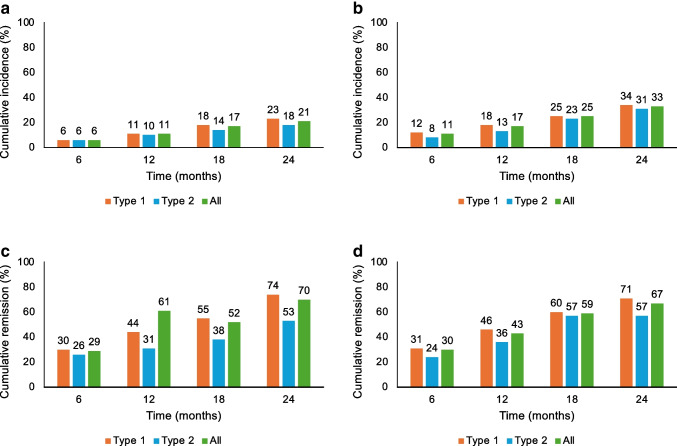
Cumulative incidence and remission (%) of elevated diabetes distress and depressive symptoms, stratified by diabetes type: (**a**) cumulative incidence of diabetes distress; (**b**) cumulative incidence of depressive symptoms; (**c**) cumulative remission of diabetes distress; (**d**) cumulative remission of depressive symptoms

Over 24 months, the cumulative incidence of elevated diabetes distress rose from 6.0% at 6 months to 21.0% at 24 months. Thus, across the 24 month analysis period, 21% of the sample newly developed elevated diabetes distress (Fig. 1a). The analysis of diabetes distress overlooked 612.8 person-years in which 78 incident cases of elevated diabetes distress were recorded. Thus, there were 12.7 new cases of elevated diabetes distress per 100 person-years.

For depressive symptoms, the cumulative incidence increased from 11.0% at 6 months to 33.0% at 24 months. Thus, nearly a third of the sample developed elevated depressive symptoms during the observation period (Fig. 1b). The analysis overlooked 514.0 person-years in which 101 incident cases were reported. Thus, there were 19.6 new cases of elevated depressive symptoms per 100 person-years.

The cumulative remission of diabetes distress rose from 29% at 6 months to 70.0% at 24 months (Fig. 1c). The cumulative remission of depressive symptoms rose from 30% at 6 months to 67.0% at 24 months (Fig. 1d). Remission rates were slightly higher among individuals with type 1 diabetes for both diabetes distress and depressive symptoms.

### Diabetes distress: Cox regression analysis of incidence and remission

**Fig. 2:**
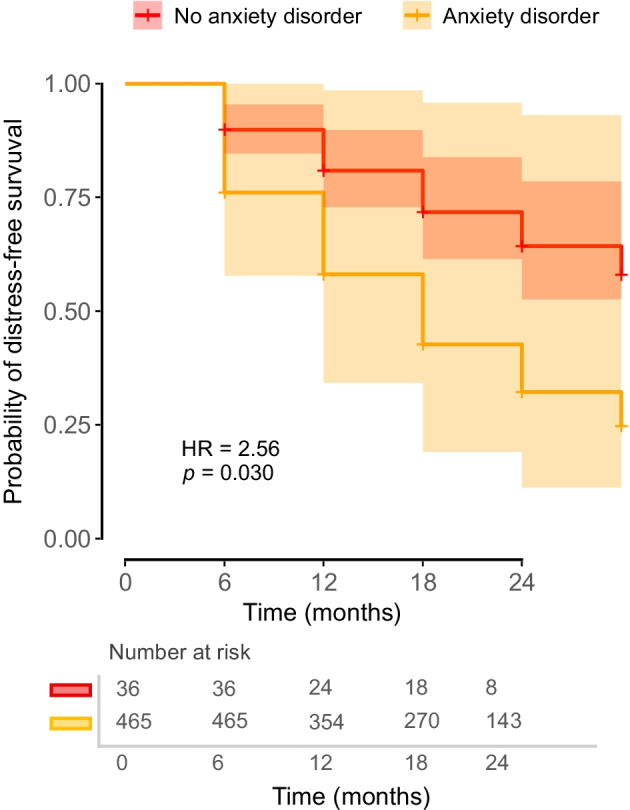
Adjusted multivariable survival curves based on a Cox proportional hazards model for the incidence of diabetes distress, stratified by the presence of an anxiety disorder. All other covariates were held constant (female gender, type 1 diabetes, absence of depression, eating disorder and complications, as well as average age, HbA_1c_ [mmol/mol] and diabetes duration)

The Cox proportional hazards model for the event of experiencing diabetes distress was built on 1622 observations. In the present analysis, fewer than 50% of participants had an incidence of diabetes distress during the observation period. The first quartile of time-to-event was 18 months, meaning that 25% of participants developed clinically relevant diabetes-related distress within the first 1.5 years of observation. The analysis revealed that HbA_1c_ levels were significantly associated with an increased risk of the event. Specifically, for each unit increase in concurrent HbA_1c_ (mmol/mol), the hazard of developing diabetes distress increased by 2% (HR=1.02 [95% CI 1.00, 1.04], *p*=0.022). In addition, gender was a significant predictor. Male participants had a lower risk of the event than female participants (HR=0.55 [95% CI 0.31, 0.98], *p*=0.043). The duration of diabetes was also significant, with longer diabetes duration associated with a slightly reduced hazard of the event (HR=0.98 [95% CI 0.95, 1.00], *p*=0.025). Furthermore, individuals with baseline long-term complications had more than double the risk of developing diabetes distress than those without complications (HR=2.11 [95% CI 1.20, 3.71], *p*=0.009). Among the baseline psychiatric comorbidities, only anxiety disorders emerged as a significant predictor, with affected individuals showing a 2.57-fold increased hazard of developing diabetes distress (HR=2.57 [95% CI 1.10, 5.99], *p*=0.029). Figure 2 depicts the adjusted survival curves for the incidence of diabetes distress, stratified by the presence of an anxiety disorder.

In comparison, the diagnosis of neither prior depression (HR=1.66 [95% CI 0.78, 3.56], *p*=0.191) nor eating disorders (HR=0.46 [95% CI 0.15, 1.37], *p*=0.164) was significantly associated with the hazard of experiencing diabetes distress. The model demonstrated good fit, with a concordance index of 0.757 (SE=0.034). The Wald test confirmed the significance of the model (χ^2^=54.69, *p*<0.001).

The Cox model for the remission of diabetes distress was built on 411 observations. The median time to remission of diabetes distress was estimated at 6 months (95% CI 6, 12). This indicates that half of the individuals in the study experienced remission of diabetes-related distress within the first 6 months, with the upper bound of the confidence interval suggesting that this time frame could extend to 12 months. None of the included predictors showed statistically significant associations with the likelihood of remission. The model’s concordance index was 0.672 (SE=0.05), suggesting a modest fit. The Wald test did not show significant evidence that the included predictors were collectively associated with remission (*p*=0.2).

The results of the Cox regression analysis for incidence and remission of diabetes distress are shown in Table 2.

**Table 2:** Multivariable Cox regression: incidence and remission of diabetes distress (PAID) and depressive symptoms (PHQ-8)

Covariate	HR (95% CI); *p* value			
	Incidence (PAID)	Remission (PAID)	Incidence (PHQ-8)	Remission (PHQ-8)
	1622 observations	411 observations	1542 observations	509 observations
HbA_1c_, mmol/mol	1.02 (1.00, 1.04); 0.022*	1.01 (0.99, 1.03); 0.400	1.03 (1.01, 1.04); 0.000***	1.00 (0.98, 1.02); 0.848
Age	0.99 (0.97, 1.01); 0.230	1.02 (1.00, 1.05); 0.116	1.00 (0.98, 1.02); 0.887	1.02 (1.00, 1.04); 0.045*
Male gender	0.55 (0.31, 0.98); 0.043*	1.09 (0.65, 1.83); 0.754	0.84 (0.53, 1.34); 0.469	0.91 (0.57, 1.45); 0.704
Type 2 diabetes	0.80 (0.38, 1.67); 0.552	0.55 (0.25, 1.19); 0.129	0.90 (0.51, 1.61); 0.734	0.70 (0.37, 1.32); 0.270
Diabetes duration	0.98 (0.95, 1.00); 0.025*	1.01 (0.99, 1.03); 0.221	0.99 (0.97, 1.01); 0.220	1.00 (0.99, 1.02); 0.963
Pre-existing long-term complications	2.11 (1.20, 3.71); 0.009*	0.85 (0.46, 1.56); 0.594	1.44 (0.92, 2.27); 0.113	0.70 (0.42, 1.16); 0.165
History of diagnosed depression	1.66 (0.78, 3.56); 0.191	0.98 (0.55, 1.77); 0.954	2.63 (1.46, 4.71); 0.001*	0.69 (0.42, 1.16); 0.162
History of diagnosed anxiety disorder	2.57 (1.10, 5.99); 0.029*	1.15 (0.59, 2.23); 0.675	1.18 (0.58, 2.40); 0.650	0.55 (0.26, 1.16); 0.118
History of diagnosed eating disorder	0.46 (0.15, 1.37); 0.164	0.43 (0.16, 1.17); 0.099	2.20 (1.02, 4.75); 0.044*	0.80 (0.36, 1.76); 0.576

^*^p<0.05, ***p<0.001

As a sensitivity analysis, we repeated the Cox regression models, replacing the dichotomous variable for prior depression diagnosis with the continuous PHQ-8 score as a time-varying variable to account for current depressive symptom burden. Notably, anxiety disorder remained a significant predictor for the incidence of diabetes distress, and higher concurrent PHQ-8 scores were associated with a lower likelihood of remission. Full model results are provided in electronic supplementary material (ESM) Tables 1 and 2.

### Depressive symptoms: Cox regression analysis of incidence and remission

**Fig. 3:**
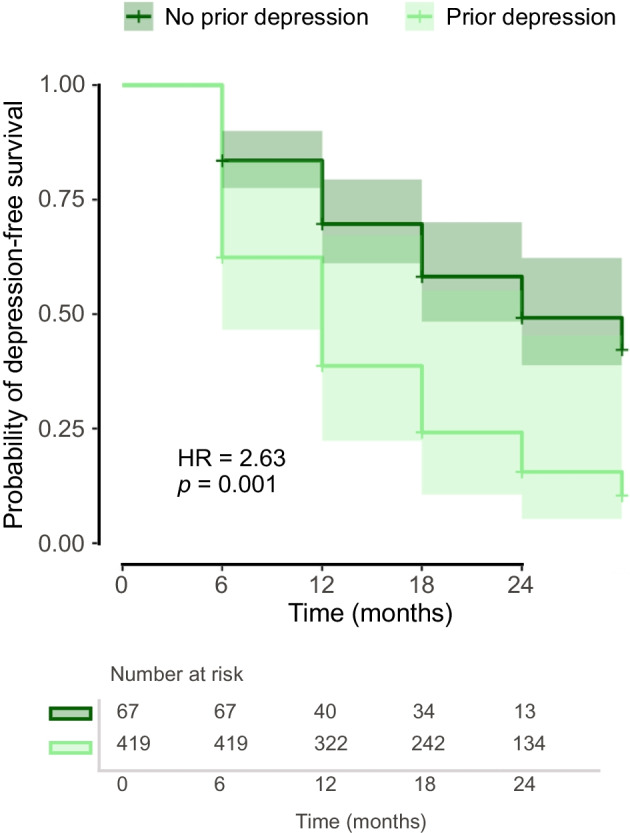
Adjusted multivariable survival curves based on a Cox proportional hazards model for the incidence of depressive symptoms, stratified by the presence of prior depression. All other covariates were held constant (female gender, type 1 diabetes, absence of depression, eating disorder and complications, as well as average age, HbA_1c_ [mmol/mol] and diabetes duration)

As regards the incidence of depressive symptoms, 1542 observations were included in the Cox model. The median survival time was determined to be 24 months, with the lower bound of the 95% CI at 18 months. Concurrent HbA_1c_ again was significantly associated with the outcome: with each unit increase in HbA_1c_ (mmol/mol), the risk of developing depressive symptoms raised by 3% (HR=1.03 [95% CI 1.01, 1.04], *p*<0.001). A prior history of depression was a strong predictor, more than doubling the risk of onset of repeated depressive symptoms (HR=2.63 [95% CI 1.46, 4.71], *p*=0.001). Figure 3 illustrates the adjusted survival curves for the incidence of depressive symptoms, stratified by the presence of prior depression. Additionally, the presence of an eating disorder significantly increased the hazard of depressive symptoms (HR=2.20 [95% CI 1.02, 4.75], *p*=0.044). Conversely, anxiety disorders were not significantly associated with the hazard of developing depressive symptoms (HR=1.18 [95% CI 0.58, 2.40], *p*=0.650). The model fit was good, with a concordance index of 0.704 (SE=0.034). The Wald test indicated that the included predictors had a significant overall effect (χ^2^=89.43, *p*<0.001).

For the remission of depressive symptoms, the Cox model included 509 observations. In the analysis, the median survival time was observed to be 6 months (95% CI 6, 6). Only age was significantly associated with the outcome. Specifically, each additional year of age increased the likelihood of remission by 2% (HR=1.02 [95% CI 1.00, 1.04], *p*=0.045). All other predictors showed no significant association. The model had a reasonable fit with a concordance index of 0.710 (SE=0.041). The Wald test was significant (χ^2^=21.17, *p*=0.001).

The results of the Cox regression analysis for incidence and remission of depressive symptoms are fully displayed in Table 2.

As a sensitivity analysis, we repeated both Cox regression models, including the continuous PAID score as a time-varying covariate to account for concurrent diabetes distress levels. Higher concurrent diabetes distress was associated with an increased risk of developing depressive symptoms and with a lower likelihood of remission. Full model results are provided in ESM Tables 3 and 4.

## Discussion

The present study provides important insights into the incidence and remission of diabetes distress and depressive symptoms in individuals with diabetes. The incidence rates observed in this study highlight the dynamic nature of these mental health outcomes, which is in line with previous research [29]. At 24 months, the cumulative incidence reached 21% for diabetes distress and 33% for depressive symptoms. In addition, remission of symptoms did not occur in every case: nearly one-third of participants showed consistently elevated levels of diabetes distress or depressive symptoms. Taken together, these findings emphasise the importance of systematically screening (every 6 months) and monitoring psychological distress and depressive symptoms as part of routine diabetes care, particularly for individuals with suboptimal glycaemic outcomes or psychiatric comorbidities [29]. This is consistent with current international guidelines such as the American Diabetes Association, which recommends regular assessment and follow-up of emotional well-being to identify emerging problems and guide treatment adjustments [38].

Using Cox proportional hazards models, we analysed time-to-event data while accounting for censored observations. Several predictors were identified as significantly associated with the onset and remission of these mental health conditions.

With regard to diabetes distress, the analysis revealed that a quarter of participants developed clinically relevant distress within the first 18 months of observation. Notably, higher HbA_1c_ levels, female gender and the presence of diabetes-related complications significantly increased the risk of developing diabetes distress. These findings are in line with previous research suggesting that suboptimal glycaemic management and diabetes-related complications are closely tied to psychological burden in diabetes [3, 5, 7–9, 12]. The protective effect of longer diabetes duration may reflect a process of psychological adaptation over time. Interestingly, anxiety disorders were the only psychiatric comorbidity significantly associated with incident diabetes distress, while prior depression and eating disorders were not.

In contrast, none of the predictors included was significantly associated with the remission of diabetes distress. Thus, no protective factors facilitating the remission of elevated diabetes distress could be identified. The median remission time was relatively short (6 months), indicating that, in our study, diabetes distress was a transient condition. However, the modest model fit suggests that additional, unmeasured factors may play a role in the resolution of distress.

Regarding depressive symptoms, the median time to onset was estimated to be 24 months, indicating that depressive symptoms emerge later than diabetes distress in the population studied. HbA_1c_ again appeared as a robust risk factor, reinforcing the notion that suboptimal metabolic outcomes are not only physiologically but also psychologically consequential, which is in line with previous research [18, 20, 30]. The strong association of prior depression with the recurrence of depressive symptoms highlights the chronic and relapsing nature of depressive disorders. Additionally, the presence of eating disorders significantly increased the hazard for depressive symptoms, reflecting an interplay between disordered eating, diabetes and mood [39–41]. In contrast with diabetes distress, anxiety disorders did not significantly predict the onset of depressive symptoms. Existing studies that used survival models, such as Cox proportional hazards regressions, yielded inconsistent findings. For example, Huang et al [31] observed, over a 1 year follow-up period, lower incidence rates of depression only among men. Similarly, Jacob and Kostev [30] found a reduced risk of depression in men with type 2 diabetes in a 10 year follow-up study. Additionally, the presence of long-term complications and elevated HbA_1c_ levels significantly increased the risk of developing depression, which is in line with our findings.

The remission of depressive symptoms was more likely in older individuals, suggesting that age may confer psychological resilience. However, no other predictors reached statistical significance. The median remission time was also 6 months.

In addition, the results challenge the notion of a linear continuum from diabetes distress to depression [2, 3]. These findings are consistent with recent network analytic research by McInerney et al [42], who demonstrated that diabetes distress, depressive symptoms and anxiety symptoms in individuals with type 2 diabetes form interrelated but distinct symptom clusters. Their analysis revealed that diabetes distress was connected to anxiety-related nodes, particularly symptoms reflecting excessive worry and difficulty relaxing. In our study, the different patterns of associations observed across outcomes support a model of distinct aetiological pathways: while anxiety disorders significantly predicted the onset of diabetes distress but not depressive symptoms, a history of depression was strongly associated with the incidence of depressive symptoms but not with diabetes distress. Therefore, the development of diabetes distress may be more closely tied to anxiety-related processes, such as worry and disease-related fears. This interpretation is supported by studies demonstrating a strong association between hypoglycaemia-related fear and elevated levels of diabetes distress [43, 44]. Depression, on the other hand, may follow a more classical mood disorder trajectory, particularly in individuals with a prior history of depressive episodes. It is crucial to understand these differences, as the clinical implication is that diabetes distress and depression could benefit from differentiated intervention strategies. Treating them as a single emotional construct could risk overlooking specific needs. Interventions for diabetes distress might include structured education or support targeting illness-related fears, whereas depressive symptoms may require approaches aligned with mood disorders.

Despite these differences, our sensitivity analyses also point to a close temporal interplay between both constructs. When including diabetes distress as a time-varying covariate, higher distress predicted the incidence and reduced the likelihood of remission of depressive symptoms. Likewise, when depressive symptoms were modelled as a time-varying covariate, it significantly predicted the course of diabetes distress. These bidirectional associations suggest that fluctuations in one condition may dynamically influence the other over time, consistent with previous longitudinal findings indicating reciprocal effects between diabetes distress and depressive symptoms [45].

A key strength of the present study is the use of Cox regression, which allowed for the modelling of time-to-event while appropriately handling censored data, which is an advantage over the traditional regression or growth models often used in similar research.

However, several limitations of this study must be considered. First, the presence of depression, anxiety disorders and eating disorders was based on self-reported lifetime history rather than current standardised diagnostic assessment, which may have led to misclassification or under-reporting. HbA_1c_ values were also self-reported. At each assessment, participants were asked to provide their most recent HbA_1c_ value, which is typically measured every 3 months as part of standard care in Germany. We therefore assume that most of the reported values reflected measurements taken within the past 3 months, although individual deviations cannot be ruled out. Second, the models did not account for potentially important time-varying covariates, such as the initiation of advanced diabetes technologies (e.g. automated insulin delivery systems) or changes in life circumstances (e.g. the incidence of long-term complications, other illnesses, career-/financial-/family-related worries and receipt of psychological treatment), which could influence both glycaemic outcomes and psychological well-being over time. Third, a methodological limitation of the survival analysis approach is that participants are censored once the event occurs, preventing the examination of subsequent fluctuations within the same analytic framework. Finally, the study sample may be subject to selection bias, as participants were drawn from a panel characterised by relatively low HbA_1c_ levels and a high degree of engagement with their diabetes management. Moreover, the sample may not be representative of the broader population of people with diabetes in Germany in terms of ethnicity, socioeconomic status, or regional distribution, as the dia·link panel primarily consists of educated, German-speaking adults who voluntarily enrol to participate in research. This may limit the generalisability of the findings to broader or more clinically vulnerable populations. Gender was included as a self-reported covariate in all analyses to examine potential gender-related differences. Male gender was associated with a lower risk of incident diabetes distress. However, as no participant identified as ‘diverse’, the analyses were restricted to comparisons between women and men and do not reflect the full complexity of gender-related influences. Future studies should aim to include more diverse samples and consider dynamic changes in both clinical and psychosocial factors across time. Furthermore, type 1 and type 2 diabetes should be analysed separately, given their differing medical and psychological contexts. In addition, further research is needed to clarify not only vulnerability factors for the incidence of elevated diabetes distress and depression, but also potential protective factors associated with the remission of this condition (e.g. resilience).

In summary, this study identifies suboptimal glycaemic outcomes (HbA_1c_) as a consistent predictor of both diabetes distress and depressive symptoms, highlighting the psychological relevance of metabolic outcomes. Anxiety disorders were specifically associated with the onset of diabetes distress, while eating disorders and a history of depression significantly predicted the incidence of depressive symptoms. These findings support the need for differentiated psychological monitoring and targeted interventions that address distinct mental health risks in people with diabetes.

## Supplementary Information

Below is the link to the electronic supplementary material.

ESM (PDF 58 KB)

## Data Availability

The datasets generated and analysed in the current study are available from the corresponding author upon reasonable request.

## Abbreviations

PAID: Problem Areas in Diabetes Scale
PHQ-8: Patient Health Questionnaire 8

## References

[1] Gonzalez JS, Tanenbaum ML, Commissariat PV (2016) Psychosocial factors in medication adherence and diabetes self-management: implications for research and practice. Am Psychol 71(7):539–551. 10.1037/a0040388 27690483 10.1037/a0040388 PMC5792162

[2] Skinner T, Joensen L, Parkin T (2020) Twenty-five years of diabetes distress research. Diabet Med 37(3):393–400. 10.1111/dme.14157 31638279 10.1111/dme.14157

[3] Snoek FJ, Bremmer MA, Hermanns N (2015) Constructs of depression and distress in diabetes: time for an appraisal. Lancet Diabetes Endocrinol 3(6):450–460. 10.1016/S2213-8587(15)00135-7 25995123 10.1016/S2213-8587(15)00135-7

[4] Perrin N, Davies M, Robertson N, Snoek F, Khunti K (2017) The prevalence of diabetes-specific emotional distress in people with Type 2 diabetes: a systematic review and meta-analysis. Diabet Med 34(11):1508–1520. 10.1111/dme.13448 28799294 10.1111/dme.13448

[5] Fisher L, Hessler DM, Polonsky WH, Mullan J (2012) When is diabetes distress clinically meaningful? Establishing cut points for the Diabetes Distress Scale. Diabetes Care 35(2):259–264. 10.2337/dc11-1572 22228744 10.2337/dc11-1572 PMC3263871

[6] Fisher L, Mullan JT, Arean P, Glasgow RE, Hessler D, Masharani U (2010) Diabetes distress but not clinical depression or depressive symptoms is associated with glycemic control in both cross-sectional and longitudinal analyses. Diabetes Care 33(1):23–28. 10.2337/dc09-1238 19837786 10.2337/dc09-1238 PMC2797978

[7] Polonsky WH, Anderson BJ, Lohrer PA et al (1995) Assessment of diabetes-related distress. Diabetes Care 18(6):754–760. 10.2337/diacare.18.6.754 7555499 10.2337/diacare.18.6.754

[8] Schmitt A, Bendig E, Baumeister H, Hermanns N, Kulzer B (2021) Associations of depression and diabetes distress with self-management behavior and glycemic control. Health Psychol 40(2):113–124. 10.1037/hea0001037 33252963 10.1037/hea0001037

[9] Schmitt A, Reimer A, Kulzer B, Haak T, Gahr A, Hermanns N (2015) Negative association between depression and diabetes control only when accompanied by diabetes-specific distress. J Behav Med 38:556–564. 10.1007/s10865-014-9604-3 25326733 10.1007/s10865-014-9604-3

[10] Joensen L, Tapager I, Willaing I (2013) Diabetes distress in type 1 diabetes – a new measurement fit for purpose. Diabet Med 30(9):1132–1139. 10.1111/dme.12241 23701311 10.1111/dme.12241

[11] Hessler D, Fisher L, Polonsky W et al (2020) There is value in treating elevated levels of diabetes distress: the clinical impact of targeted interventions in adults with type 1 diabetes. Diabet Med 37(1):71–74. 10.1111/dme.14082 31314907 10.1111/dme.14082

[12] Fisher L, Polonsky W, Hessler D (2019) Addressing diabetes distress in clinical care: a practical guide. Diabet Med 36(7):803–812. 10.1111/dme.13967 30985025 10.1111/dme.13967

[13] Farooqi A, Gillies C, Sathanapally H et al (2022) A systematic review and meta-analysis to compare the prevalence of depression between people with and without type 1 and type 2 diabetes. Prim Care Diabetes 16(1):1–10. 10.1016/j.pcd.2021.11.001 34810141 10.1016/j.pcd.2021.11.001

[14] Schram MT, Baan CA, Pouwer F (2009) Depression and quality of life in patients with diabetes: a systematic review from the European depression in diabetes (EDID) research consortium. Curr Diabetes Rev 5(2):112–119. 10.2174/157339909788166828 19442096 10.2174/157339909788166828 PMC2764861

[15] Gonzalez JS, Peyrot M, McCarl LA et al (2008) Depression and diabetes treatment nonadherence: a meta-analysis. Diabetes Care 31(12):2398–2403. 10.2337/dc08-1341 19033420 10.2337/dc08-1341 PMC2584202

[16] Nouwen A, Adriaanse M, van Dam K et al (2019) Longitudinal associations between depression and diabetes complications: a systematic review and meta-analysis. Diabet Med 36(12):1562–1572. 10.1111/dme.14054 31215077 10.1111/dme.14054

[17] Beran M, Muzambi R, Geraets A et al (2022) The bidirectional longitudinal association between depressive symptoms and HbA1c: a systematic review and meta-analysis. Diabet Med 39(2):e14671. 10.1111/dme.14671 34407250 10.1111/dme.14671 PMC9292323

[18] Hermanns N, Kulzer B, Krichbaum M, Kubiak T, Haak T (2005) Affective and anxiety disorders in a German sample of diabetic patients: prevalence, comorbidity and risk factors. Diabet Med 22(3):293–300. 10.1111/j.1464-5491.2005.01414.x 15717877 10.1111/j.1464-5491.2005.01414.x

[19] Roy T, Lloyd CE (2012) Epidemiology of depression and diabetes: a systematic review. J Affect Disord 142:8–21. 10.1016/S0165-0327(12)70004-6 10.1016/S0165-0327(12)70004-6 23062861

[20] Téllez-Zenteno JF, Cardiel MH (2002) Risk factors associated with depression in patients with type 2 diabetes mellitus. Arch Med Re 33(1):53–60. 10.1016/S0188-4409(01)00349-6 10.1016/s0188-4409(01)00349-6 11825632

[21] Fisher L, Gonzalez J, Polonsky W (2014) The confusing tale of depression and distress in patients with diabetes: a call for greater clarity and precision. Diabet Med 31(7):764–772. 10.1111/dme.12428 24606397 10.1111/dme.12428 PMC4065190

[22] Young-Hyman D, de Groot M, Hill-Briggs F, Gonzalez JS, Hood K, Peyrot M (2017) Psychosocial care for people with diabetes: a position statement of the American Diabetes Association. Diabetes Care 40(2). 10.2337/dc17-er02 10.2337/dc17-er02 PMC5267313 27927693

[23] Fisher L, Mullan JT, Skaff MM, Glasgow RE, Arean P, Hessler D (2009) Predicting diabetes distress in patients with type 2 diabetes: a longitudinal study. Diabet Med 26(6):622–627. 10.1111/j.1464-5491.2009.02730.x 19538238 10.1111/j.1464-5491.2009.02730.x PMC2740749

[24] Lipscombe C, Burns RJ, Schmitz N (2015) Exploring trajectories of diabetes distress in adults with type 2 diabetes; a latent class growth modeling approach. J Affect Disord 188:160–166. 10.1016/j.jad.2015.08.003 26363265 10.1016/j.jad.2015.08.003

[25] Fisher L, Hessler D, Polonsky W, Strycker L, Masharani U, Peters A (2016) Diabetes distress in adults with type 1 diabetes: prevalence, incidence and change over time. J Diabetes Complications 30(6):1123–1128. 10.1016/j.jdiacomp.2016.03.032 27118163 10.1016/j.jdiacomp.2016.03.032 PMC4949147

[26] Kampling H, Petrak F, Farin E, Kulzer B, Herpertz S, Mittag O (2017) Trajectories of depression in adults with newly diagnosed type 1 diabetes: results from the German Multicenter Diabetes Cohort Study. Diabetologia 60:60–68. 10.1007/s00125-016-4123-0 27787619 10.1007/s00125-016-4123-0

[27] Whitworth S, Bruce DG, Starkstein S et al (2017) Depression symptoms are persistent in type 2 diabetes: risk factors and outcomes of 5-year depression trajectories using latent class growth analysis. Diabet Med 34(8):1108–1115. 10.1111/dme.13372 28453875 10.1111/dme.13372

[28] De Groot M, Crick KA, Long M, Saha C, Shubrook JH (2016) Lifetime duration of depressive disorders in patients with type 2 diabetes. Diabetes Care 39(12):2174–2181. 10.2337/dc16-1145 27729427 10.2337/dc16-1145 PMC5127229

[29] Nefs G, Pouwer F, Denollet J, Pop V (2012) The course of depressive symptoms in primary care patients with type 2 diabetes: results from the Diabetes, Depression, Type D Personality Zuidoost-Brabant (DiaDDZoB) Study. Diabetologia 55(3):608–616. 10.1007/s00125-011-2411-2 22198261 10.1007/s00125-011-2411-2 PMC3268983

[30] Jacob L, Kostev K (2016) Prevalence of depression in type 2 diabetes patients in German primary care practices. J Diabetes Complications 30(3):432–437. 10.1016/j.jdiacomp.2015.12.013 26790576 10.1016/j.jdiacomp.2015.12.013

[31] Huang C-J, Lin C-H, Lee M-H, Chang K-P, Chiu H-C (2012) Prevalence and incidence of diagnosed depression disorders in patients with diabetes: a national population-based cohort study. Gen Hosp Psychiatry 34(3):242–248. 10.1016/j.genhosppsych.2011.12.011 22325626 10.1016/j.genhosppsych.2011.12.011

[32] de Wit M, Pouwer F, Snoek F (2022) How to identify clinically significant diabetes distress using the Problem Areas in Diabetes (PAID) scale in adults with diabetes treated in primary or secondary care? Evidence for new cut points based on latent class analyses. BMJ Open 12(3):e056304. 10.1136/bmjopen-2021-056304 35277408 10.1136/bmjopen-2021-056304 PMC8919470

[33] de la Torre JA, Vilagut G, Ronaldson A et al (2023) Reliability and cross-country equivalence of the 8-item version of the Patient Health Questionnaire (PHQ-8) for the assessment of depression: results from 27 countries in Europe. Lancet Reg Health Eur 31:100659. 10.1016/j.lanepe.2023.100659 37332385 10.1016/j.lanepe.2023.100659 PMC10272490

[34] Katon WJ, Simon G, Russo J et al (2004) Quality of depression care in a population-based sample of patients with diabetes and major depression. Med Care 42(12):1222–1229. 10.1097/00005650-200412000-00009 15550802 10.1097/00005650-200412000-00009

[35] R Core Team (2022) R: A language and environment for statistical computing. R Foundation for Statistical Computing. Available from https://www.R-project.org/. Accessed 16 Oct 2025

[36] George B, Seals S, Aban I (2014) Survival analysis and regression models. J Nucl Cardiol 21(4):686–694. 10.1007/s12350-014-9908-2 24810431 10.1007/s12350-014-9908-2 PMC4111957

[37] Allison PD (2018) Event history and survival analysis. In: Hancock GR, Stapleton LM, Mueller RO (eds) The reviewer’s guide to quantitative methods in the social sciences. Routledge, New York, pp 86–97

[38] American Diabetes Association (2024) 2. Diagnosis and Classification of Diabetes: Standards of Care in Diabetes—2024. Diabetes Care 47(Supplement_1):20–42. 10.2337/dc24-S002 10.2337/dc24-S002 PMC10725812 38078589

[39] Bächle C, Lange K, Stahl-Pehe A et al (2015) Symptoms of eating disorders and depression in emerging adults with early-onset, long-duration type 1 diabetes and their association with metabolic control. PLoS One 10(6):e0131027. 10.1371/journal.pone.0131027 26121155 10.1371/journal.pone.0131027 PMC4487688

[40] Ripoli C, Ricciardi MR, Zuncheddu E, Angelo MR, Pinna AP, Ripoli D (2022) Emotional eating and disordered eating behaviors in children and adolescents with type 1 diabetes. Sci Rep 12(1):21854. 10.1038/s41598-022-26271-2 36528643 10.1038/s41598-022-26271-2 PMC9759523

[41] Celik S, Kayar Y, Akçakaya RÖ et al (2015) Correlation of binge eating disorder with level of depression and glycemic control in type 2 diabetes mellitus patients. Gen Hosp Psychiatry 37(2):116–119. 10.1016/j.genhosppsych.2014.11.012 25670634 10.1016/j.genhosppsych.2014.11.012

[42] McInerney AM, Lindekilde N, Nouwen A, Schmitz N, Deschênes SS (2022) Diabetes distress, depressive symptoms, and anxiety symptoms in people with type 2 diabetes: a network analysis approach to understanding comorbidity. Diabetes Care 45(8):1715–1723. 10.2337/dc21-2297 35704532 10.2337/dc21-2297

[43] Hapunda G, Abubakar A, Pouwer F, Van De Vijver F (2020) Correlates of fear of hypoglycemia among patients with type 1 and 2 diabetes mellitus in outpatient hospitals in Zambia. Int J Diabetes Dev Ctries 40(4):619–626. 10.1007/s13410-020-00835-2

[44] Li S, Fang L, Lee A et al (2021) The association between diabetes-related distress and fear of hypoglycaemia in patients with type 2 diabetes mellitus: a cross-sectional descriptive study. Nursing Open 8(4):1668–1677. 10.1002/nop2.800 33605564 10.1002/nop2.800 PMC8186714

[45] Ehrmann D, Kulzer B, Haak T, Hermanns N (2015) Longitudinal relationship of diabetes-related distress and depressive symptoms: analysing incidence and persistence. Diabet Med 32(10):1264–1271. 10.1111/dme.12861 26202341 10.1111/dme.12861

