# Supplementary material for: Uncovering distinct predictors of diabetes distress and depressive symptoms in a longitudinal survival analysis of incidence and remission: indication for diverging aetiological paths
Source: Diabetologia. 2026 Jan 22;69(5):1205–15. doi: 10.1007/s00125-026-06666-w (PMC13005816; doi:10.1007/s00125-026-06666-w)
Supplement: Supplementary file 1 — ESM (PDF 58 KB) [file 125_2026_6666_MOESM1_ESM.pdf]

ESM

Diabetes distress: Cox regression analysis of incidence and remission

As a sensitivity analysis, we repeated the Cox regression model predicting the incidence of diabetes distress, replacing the dichotomous variable for prior depression diagnosis with the continuous PHQ-8 score as a time-varying variable to account for current depressive symptom burden. Notably, anxiety disorder remained a significant predictor (HR=2.28 [95% CI 1.14–4.55], p=0.020), despite the high conceptual overlap between PHQ-8 and diabetes distress. These results suggest that anxiety may contribute independently to the development of diabetes distress, beyond the effects of concurrent depressive symptoms. For full model results, see ESM Table 1. We did the same sensitivity analysis for the remission of diabetes distress, in which higher concurrent PHQ-8 scores were associated with a lower likelihood of remission (HR=0.90 [95% CI 0.87–0.94], p<0.001), while the remaining results were comparable to the original model. For full model results, see ESM Table 2.

ESM Table 1

Cox Regression: Incidence of Diabetes Distress using continuous PHQ-8 as covariate

**Covariate**	***HR* [95% CI]; *p***
HbA1c (mmol/mol)	1.02 (1.00–1.04); 0.126
Age	0.99 (0.97–1.01); 0.463
Male gender	0.62 (0.35–1.11); 0.109
Type 2 diabetes	0.78 (0.37–1.63); 0.507
Diabetes duration	0.98 (0.96–1.00); 0.071
Long-term complications	1.61 (0.92–2.81); 0.094
**PHQ-8**	**1.13 (1.08–1.18); 0.000****
**History of diagnosed anxiety disorder**	**2.28 (1.14–4.55); 0.020***
History of diagnosed eating disorder	0.35 (0.11–1.13); 0.080

Cox regression results for incidence and remission of depressive symptoms, * $p < 0.05$, ** $p < 0.001$. Concordance = 0.801 (*SE* = 0.026). Wald test $\chi^2 = 102.0$, $p < .001$.

ESM Table 2

Cox Regression: Remission of Diabetes Distress using continuous PHQ-8 as covariate

**Covariate**	***HR* [95% CI]; *p***
HbA1c (mmol/mol)	1.02 (1.00–1.04); 0.086
Age	1.02 (0.99–1.04); 0.129
Male gender	1.23 (0.75–2.02); 0.420
Type 2 diabetes	0.55 (0.25–1.17); 0.121
Diabetes duration	1.01 (1.00–1.03); 0.089
Long-term complications	0.74 (0.41–1.32); 0.305
**PHQ-8**	**0.90 (0.87–0.94); 0.000****
History of diagnosed anxiety disorder	1.26 (0.71–2.21); 0.429
History of diagnosed eating disorder	0.62 (0.24–1.58); 0.317

Cox regression results for incidence and remission of depressive symptoms, * $p < 0.05$, ** $p < 0.001$. Concordance = 0.756 (*SE* = 0.041). Wald test $\chi^2 = 44.61$, $p < .001$.

Depressive symptoms: Cox regression analysis of incidence and remission

As a sensitivity analysis, we repeated both Cox regression models predicting the incidence and remission of depressive symptoms, including the continuous PAID score as a time-varying covariate to account for concurrent diabetes distress levels. In the incidence model, higher concurrent diabetes distress was significantly associated with an increased risk of developing depressive symptoms (HR=1.03 [95% CI 1.02–1.04], p<0.001). Similarly, in the remission model, higher concurrent diabetes distress predicted a lower likelihood of remission (HR=0.98 [95% CI 0.97–0.99], p<0.001). The remaining predictors showed patterns comparable to the original analyses. Full model results are provided in ESM Tables 3 and 4.

ESM Table 3

Cox Regression: Incidence of Depressive Symptoms using continuous PAID as covariate

**Covariate**	***HR* [95% CI]; *p***
**HbA1c (mmol/mol)**	**1.02 (1.00–1.04); 0.016***
Age	1.00 (0.99–1.02); 0.687
Male gender	0.97 (0.61–1.54); 0.896
Type 2 diabetes	1.02 (0.57–1.80); 0.956
Diabetes duration	1.00 (0.98–1.01); 0.872
Long-term complications	1.24 (0.78–1.99); 0.367
**PAID**	**1.03 (1.02–1.04); 0.000****
**History of diagnosed depression**	**2.18 (1.24–3.82); 0.007***
History of diagnosed anxiety disorder	0.95 (0.46–1.94); 0.880
**History of diagnosed eating disorder**	**2.06 (1.01–4.23); 0.048***

Cox regression results for incidence and remission of depressive symptoms, * $p < 0.05$, ** $p < 0.001$. Concordance = 0.756 (*SE* = 0.028). Wald test $\chi^2 = 149.6$, $p < .001$.

ESM Table 4

Cox Regression: Remission of Depressive Symptoms using continuous PAID as covariate

**Covariate**	***HR* [95% CI]; *p***
HbA1c (mmol/mol)	1.00 (0.99–1.02); 0.803
**Age**	**1.02 (1.00–1.04); 0.043***
Male gender	0.90 (0.59–1.38); 0.634
Type 2 diabetes	0.62 (0.33–1.17); 0.138
Diabetes duration	1.00 (0.98–1.01); 0.525
Long-term complications	0.77 (0.47–1.27); 0.305
**PAID**	**0.98 (0.97–0.99); 0.000****
History of diagnosed depression	0.79 (0.48–1.30); 0.356
History of diagnosed anxiety disorder	0.54 (0.26–1.12); 0.098
History of diagnosed eating disorder	0.83 (0.39–1.78); 0.634

Cox regression results for incidence and remission of depressive symptoms, * $p < 0.05$, ** $p < 0.001$. Concordance = 0.747 (*SE* = 0.038). Wald test $\chi^2 = 41.85$, $p < .001$.
